# Psychotherapy training in postgraduate psychiatry training in Nigeria – Are we doing enough?

**DOI:** 10.1017/gmh.2024.32

**Published:** 2024-03-21

**Authors:** Frances Nkechi Adiukwu, Oluwadolapo Olujinmi Adedapo, Margaret Isioma Ojeahere, Umar Baba Musami, Mohammed Yusuf Mahmood, Amina Saidu Kakangi, Mumeen Olaitan Salihu, Mariana Pinto da Costa

**Affiliations:** 1 Department of Mental Health, College of Health Sciences, University of Port Harcourt, Rivers State, Nigeria; 2 Department of Neuropsychiatry, University of Port Harcourt Teaching Hospital, Rivers State, Nigeria; 3 Assessment/Psychiatric Intensive Care Unit, Neuropsychiatric Hospital, Aro, Nigeria; 4 Department of Psychiatry, Jos University Teaching Hospital, Jos, Nigeria; 5 Mental Health Department, University of Maiduguri, Maiduguri, Nigeria; 6 Federal Neuropsychiatric Hospital, Maiduguri, Nigeria; 7 Mental Health Department, Federal Medical Centre Abuja, Nigeria; 8 Department of Behavioural Sciences, University of Ilorin Teaching Hospital, Ilorin, Nigeria; 9 South London and Maudsley NHS Foundation Trust, London, UK; 10 Institute of Psychiatry, Psychology & Neuroscience, King’s College London, London, UK; 11 Institute of Biomedical Sciences Abel Salazar, University of Porto, Porto, Portugal

**Keywords:** early career psychiatrists, psychotherapy training, psychiatry trainees, psychotherapy, Nigeria

## Abstract

**Introduction:**

Nigeria, with an estimated 40 million people living with mental disorders, faces a critical shortage of psychiatrists to address the significant burden. Despite psychotherapy being integral to psychiatric training, it lacks structure, consistency and adequate supervision. This is alarming, particularly given the substantial demand for specialized psychotherapeutic support among those with mental illness.

**Methodology:**

A cross-sectional study utilised an online survey targeting early career psychiatrists recruited from the Early Career Psychiatrists section of the Association of Psychiatrists in Nigeria.

**Results:**

The questionnaire was distributed to 252 early career psychiatrists across Nigeria, with a 42.9% response rate, of which 50% were male, with 73.2% trainees and 26.8% early career psychiatrists. While 52.8% had received some psychotherapy training, only 2.8% were qualified psychotherapists. Notably, only 27.6% of those with psychotherapy training had over 100 hours of training. Factors such as receiving psychotherapy training during postgraduate training and having supervised psychotherapy training were found significant for having independent psychotherapy training.

**Conclusions:**

There is little emphasis on psychotherapy training in the postgraduate training in Nigeria. Those who had full training in psychotherapy were self-sponsored. Structured, supervised and mandatory psychotherapy within psychiatry training curriculum is recommended.

## Graphical abstract

**Figure:**
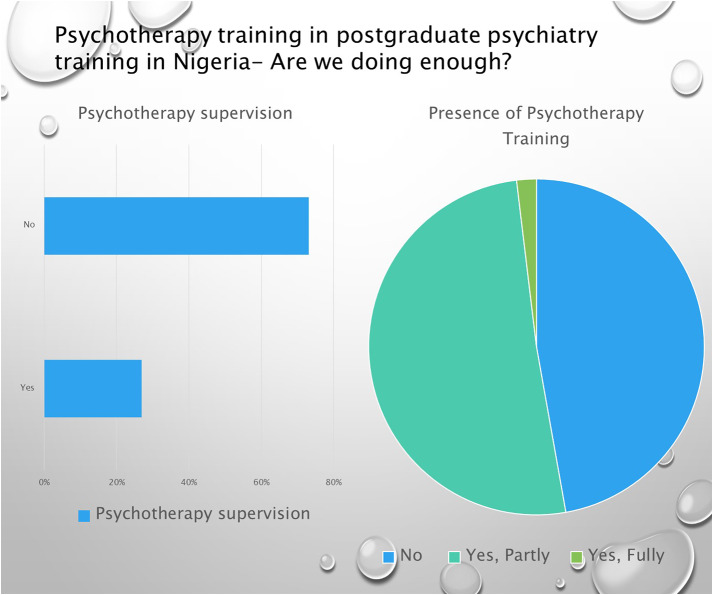

## Impact statement

Global best practices promote the training of psychiatry trainees in various techniques of psychotherapy and its incorporation into the training curriculum, hence the need to assess the extent and factors associated with the completion of psychotherapy training among early career psychiatrists (ECPs). Almost half of the ECPs in Nigeria had some form of psychotherapy training, however, only a few had full psychotherapy training. This study reveals the need for the inclusion of mandatory psychotherapy in the current psychiatry training curriculum, which will consequently improve psychiatric practice and decrease the treatment gap in mental healthcare. This inclusion will go a long way in improving the quality of holistic care delivered to mental health patients by ECPs.

## Introduction

Nigeria is a low and middle-income country in West Africa with an estimated population of 40 million people living with mental disorders, which span across mild to severe forms (Lansana, 2017). Interventions to support people with mental disorders may require psychotherapy alone or in combination with other biomedical therapies (Madu, 2016). Despite the high prevalence of mental disorders, Nigeria has only about 250 Nigerian-trained and qualified psychiatrists, 200 psychiatry trainees, and 319 licensed clinical psychologists (Bakare, 2021; Coker, 2022). As part of their clinical education, psychiatric trainees should be well-equipped with psychotherapeutic techniques. Psychotherapy is a useful therapeutic resource that, over time, has evolved and expanded, and is currently considered a mainstay of treatment for mental disorders (Locher et al., 2019). Although recent advances in biological psychiatry and neuroscience have put pharmacotherapy at the forefront giving it more clinical consideration, psychotherapy remains a widely accepted and evidence-based form of treatment for mental disorders (Tavakoli, 2014). Psychiatrists and psychiatry trainees have a critical role in the management of individuals with mental disorders, as they represent a significant part of mental healthcare providers capable of providing integrative pharmacotherapy and psychotherapy (Locher et al., 2019). Despite this proviso, a considerable number of psychiatrists seem to lack skills in specialized psychotherapeutic techniques and seem to shy away from conducting this form of intervention (Madu, 2016).

According to the Specialist Training General Psychiatry curriculum of the National Postgraduate Medical College of Nigeria, the minimum duration of psychiatry training is 4 years. Successfully passing the primary examination qualifies a doctor to commence residency training. The junior residency training is expected to last a minimum of 24 months, and after passing the part 1 examination, trainees proceed to senior residency training for another 24 months, at the minimum. At the end of the senior residency training, the part 2 examinations are written, and successful trainees are awarded the College Fellowship in Psychiatry (FMCPsych).

To qualify for the part 1 examination, a minimum of 6 months rotation in adult psychiatry, 3 months rotation in neurology, and 3 months in each of any 5 out of the 7 following core rotation areas is required: child psychiatry, psychiatry of later life, forensic psychiatry, community psychiatry, substance use psychiatry, emergency psychiatry and consultation-liaison psychiatry. During the senior residency training, the two outstanding core rotations are completed in 6 months, 3 months rotation in a subspecialty area apart from intended subspecialization, 3 months elective rotation and the remaining 12 months is expected to be in the area of intended subspecialization. Although the curriculum incorporates psychotherapy training during the postgraduate training, it does not include psychotherapy as one of the mandatory rotations and it is unclear how well this is supervised, considering the limited number of psychotherapists and psychologists within the country (NPMCN, 2018). Psychotherapy is mostly conducted by psychologists. Previously, concerns have been raised that psychiatrists who lack psychotherapeutic skills will be unable to offer appropriate care to the burgeoning number of individuals with mental disorders if all they continue to offer is pharmacotherapy (Holmes et al., 2007).

In Nigeria, the National Postgraduate Medical College of Nigeria and the West Africa College of Physicians are the two recognized institutions that coordinate and superintend postgraduate medical education programs (Okonofua, 2018). Postgraduate psychiatry training has existed in Nigeria for over four decades. Nigeria has over 20 postgraduate psychiatry training centers with variations in the exposure to clinical psychotherapy training due to the absence of a consistent psychotherapy evaluation program beyond course modules. Psychotherapy was incorporated into the junior postgraduate psychiatry training in Nigeria, but the program does not address the exact duration of psychotherapy training within the first 24 months period for junior residency (NPMCN, 2018).

Little is known about psychotherapy training in Nigeria, leading to the dire need to assess the views and experiences of ECPs in Nigeria on their psychotherapy curriculum and training.

To address this, this study aimed to investigate the access to, and experiences with, psychotherapy in the various training centers across Nigeria, namely:

- To determine the extent to which psychotherapy training is included in psychiatry training programs in Nigeria;
- To determine the factors associated with the completion of psychotherapy training by psychiatric trainees in Nigeria.

## Methods

This has been a cross-sectional study, part of a larger international study, the World Psychotherapy Survey. Study participants were ECPs recruited from the database (containing the names and contact details) of the ECP section of the Association of Psychiatrists in Nigeria (APN). In Nigeria, ECP is defined as psychiatry trainees (those currently undertaking residency training) and psychiatrists within 5 years post qualification from both postgraduate training colleges (National Postgraduate Medical College of Nigeria, Faculty of Psychiatry; West African College of Physicians, Faculty of Psychiatry). There is no age criteria in this definition.

The questionnaire was distributed via email to ECPs between November 2021 and February 2022 through a google form. The questionnaire was developed for the World Psychotherapy Survey conducted by the Early Career Psychiatrists Section of the World Psychiatric Association (Eissazade et al., 2021; Rai et al., 2021; Belinati Loureiro et al., 2023; Kaya et al., 2023). The self-administered questionnaire has 16 items covering three areas: i) socio-demographics, ii) psychotherapy training (types and modalities) and iii) satisfaction with psychotherapy training.

Participation in the survey was voluntary, and all information was kept confidential.

Data was transferred to the statistical package for social sciences version 20 (SPSS 20) for data analysis. Descriptive statistics were used to report the socio-demographics of the study participants, types and modalities of psychotherapy training received and training satisfaction. Non-parametric tests (chi-square and Fisher’s exact) were used to determine factors associated with the modalities of psychotherapy training.

## Results

The survey was distributed to 252 members of the ECP section of the APN. One hundred and eight participants submitted the completed survey form, giving a response rate of 42.9%. Fifty percent of the participants were males and 50% were females. The majority, 56.7% (*n* = 59) of participants, were within the 36–45 years of age bracket. Among the professionals, psychiatry trainees accounted for 73.2% (*n* = 79), and the psychiatrists within their first 5 years after training for 26.8% (*n* = 29). Of the study participants, most (*n* = 86, 79.6%) reported awareness of the inclusion of psychotherapy training during the residency training program in Nigeria and most (*n* = 60, 71.4%) reported psychotherapy training as mandatory. More than half (*n* = 57, 52.8%) of participants had psychotherapy training, whether completed or partial (which is the case in the majority of the cases), during their residency training period (during junior residency training where it is included in their training curriculum), and only three (2.8%) completed psychotherapy training during residency (Table 1).

**Table 1.** Participants socio-demographics and psychotherapy training experience

	*n*	*%*
Gender		
Female	54	50
Male	54	50
Total	108	100
Age		
29–35	41	39.4
36–45	59	56.7
46–54	4	3.9
Total	104	100
Type of professional		
Psychiatry trainee	79	73.2
Adult psychiatrist	23	21.3
Child and adolescent psychiatrist	4	3.7
Community/consultation liaison psychiatry	1	0.9
NR	1	0.9
Total	108	100
Awareness of the inclusion of psychotherapy training during postgraduate training		
No	22	20.4
Yes	86	79.6
Total	108	100
If yes		
Mandatory	60	71.4
Optional	24	28.6
Total	84	100
Who funds psychotherapy training?		
Fully by the trainee	61	56.5
Fully by the hospital/training institution	21	19.5
Fully by the government	15	13.9
Part by the trainee and part from other sources	10	9.3
NR	1	0.9
Total	108	100
Have you had any psychotherapy training?		
No	51	47.2
Yes, currently training	54	50
Yes, completed training	3	2.8
Total	108	100

NR: Not responded.

Where psychotherapy training was available during the junior residency training program, it was fully funded by the participants in more than half (*n* = 61, 56.5%) of the cases. Psychotherapy training was given in theory in the majority of the cases, with cognitive behavioral therapy being the most taught form of psychotherapy (Table 2).

**Table 2.** Details on the format of training

	*n*	%
How training was provided		
Theoretical teaching on psychotherapy	58	68.24
Theoretical teaching on psychotherapy and practical psychotherapy training	16	18.82
Practical psychotherapy	7	8.24
Theoretical teaching on psychotherapy and 1other	2	2.35
Practical psychotherapy training and 1 other	1	1.18
Theoretical teaching on psychotherapy and 2 others	1	1.18
Total	85	100
Types of psychotherapy techniques trained in		
Cognitive behavioral therapy and 2 other types of techniques	26	31.33
Cognitive behavioral therapy and 3 other types of techniques	23	27.71
Cognitive behavioral therapy	10	12.05
Cognitive behavioral therapy, family therapy	9	10.84
Cognitive behavioral therapy and 4 other types of techniques	5	6.02
Family therapy	5	6.02
Cognitive behavioral therapy, psychodynamic	2	2.41
Family therapy, Interpersonal therapy	2	2.41
Interpersonal therapy	1	1.2
Total	83	100
Satisfaction with training		
Very satisfied	6	10.53
Satisfied	20	35.09
Neither satisfied nor dissatisfied	19	33.33
Dissatisfied	9	15.79
Very dissatisfied	3	5.26
Total	57	100

### Psychotherapy supervision

Of the participants who received psychotherapy training, about one-third received supervision for their training. To these, training supervision was mandatory for only 48% (*n* = 14) of the participants. Training was mostly given in groups and lasted less than 50 hours in total (Table 3).

**Table 3.** Psychotherapy supervision

	*n*	%
Psychotherapy training supervision available		
No	79	73.15
Yes	29	26.85
Total	108	100
Mandatory supervision		
Yes	14	48.28
No	15	51.72
Total	29	100
Format of supervision		
In groups	11	37.93
Individual	7	24.14
Individual and in groups	11	37.93
Total	29	100
Duration of training		
50–100 h	7	24.14
<50 h	14	48.28
>100 h	8	27.59
Total	29	100

### Factors associated with mandatory psychotherapy training

Where psychotherapy training was mandatory, it also tended to have optional supervision (*p* = 0.027), which was funded by the participants (*p* = 0.002). Other factors such as the psychiatry specialty, type of psychotherapy training available, and whether training was theoretical or practical did not have any association with the training being either mandatory or optional (Table 4).

**Table 4.** Factors associated with mandatory psychotherapy training

Variables	Mandatory psychotherapy training	Optional psychotherapy training	*F*	*p*
Types of training				
Theoretical	42 (70.0)	18 (30.0)	6.715	0.243^F^
Practical	7 (100.0)	0		
Personal	0	0		
Theoretical/practical	12 (75.0)	4 (25.0)		
Personal/practical	0	1(100.0)		
All three	2 (100.0)	0		
Total	63	23		
Types of Psychotherapy available				
Cognitive behavioral therapy (CBT)	6 (66.7)	3 (33.3)	2.105	0.777^F^
Family therapy (FT)	4 (80.0)	1 (20.0)		
Interpersonal therapy (IT)	1 (100.)	0		
CBT and FT	8 (61.5)	5 (38.5)		
3 or more types	44 (75.9)	14 (24.1)		
Total	63	23		
Nature of psychotherapy supervision				
Mandatory	14 (51.9)	13 (48.1)	5.40	0.027^F^*
Optional	0 (0.0)	6 (100.0)		
Total	14	19		
Nature of sponsorship				
Fully self–sponsored	27 (57.4)	20 (42.6)	13.45	0.002^F^*
Fully by training institution	16 (100.0)	0(0.0)		
Fully by government	10(90.9)	1 (9.1)		
Partly by trainee and partly by other sources	6 (66.7)	3 (33.3)		
Others	1 (100.0)	0		
Total	60	24		

*F*, Fisher’s exact test.

## Discussion

### Main findings

Despite over half of the participants stating that psychotherapy training was included in their psychiatry postgraduate training and as a mandatory requirement, just about half received some form of psychotherapy training, and only 3% of the participants in Nigeria had full psychotherapy training. This might be a result of this mandatory training being self-funded as well as being theoretical in nature in the majority of the cases.

Despite the relatively low number, all qualified psychotherapists undertook self-sponsored psychotherapy training. Mandatory psychotherapy training was associated with it being self-funded and mandatory supervision of the training. Despite this, the completion rate was poor, indicating that priority was not being given to psychotherapy training in Nigerian psychiatry postgraduate training. These findings also show a general dissatisfaction with the psychotherapy training received during the training period.

### Comparison with other literature

There is generally less emphasis on psychotherapy training during postgraduate training in Nigeria than in other countries, as it is not mandatory in the curriculum (Jain et al., 2012).

The findings from this study in Nigeria are consistent with the results from this survey in Nepal, where one-third of ECPs received psychotherapy training (Rai et al., 2021). The present study also observed that psychotherapy training varies in different training institutions and was not made mandatory, with low supervision rates. However, findings from this survey in Iran reported that almost all participants had mandatory psychotherapy training (Eissazade et al., 2021). While there are a lot of constraints in psychotherapy training during psychiatry training, which includes poor funding and supervision, over half of the respondents in Nigeria sponsored their own psychotherapy training. This is similar to the results from a psychotherapy survey conducted across Europe, where trainees reported self-funding for psychotherapy training (Giacco et al., 2012). Nonetheless, many participants in Nigeria considered that the payment for the psychotherapy training should not be done by the participants themselves and wished the current training be improved upon through full incorporation and implementation of a uniform, mandatory, well-supervised accredited psychotherapy curriculum during postgraduate training in line with global best practices. Evidence suggests that online psychotherapy training provides equal knowledge, competency and confidence in psychotherapy when compared with supervised training. This, therefore, may create an option for a cheaper form of training that can be undertaken by psychiatry trainees and ECPs in low- and middle-income countries (Bennett-Levy et al., 2020).

### Implications of the findings for practice, policies and research

While there are different psychotherapy modalities, psychotherapy training should focus on common models such as cognitive behavioral therapy, family therapy and interpersonal therapy as well as group and individual therapy techniques. In Nigeria, psychiatry trainees and ECPs undergoing psychotherapy training are exposed to the common models listed above where such training exists. There is a need for the current psychiatry postgraduate training curriculum to make psychotherapy training mandatory and ensure adequate supervision as a requirement for qualification as a psychiatrist. Increased attention should be given to psychotherapy training in the postgraduate psychiatry training program curriculum. This will improve psychiatric practice and decrease the treatment gap that exists in mental health care.

### Strengths and limitations

This is the first study to investigate psychotherapy training in Nigeria; however, its limitations are the relatively low response rate and small sample size. This affects the power of the study and the reliability of the measures of associations used to determine factors affecting psychotherapy training.

## Conclusions

Psychotherapy training offered during psychiatry postgraduate training, though present in the postgraduate curriculum, lacks structure and mandatory supervision and is not mandatory for qualification as a psychiatrist in Nigeria. ECPs are largely unsatisfied with the training and tend to seek further training outside of the postgraduate training. Despite this, very few are qualified psychotherapists. This highlights the need to improve the delivery of the training curriculum as it pertains to psychotherapy training so as to decrease the treatment gap in psychiatry present in Nigeria.

## Data Availability

The data used to support the findings of this study are included within the article.
