# Supplementary material for: Psychotherapy training in postgraduate psychiatry training in Nigeria – Are we doing enough?
Source: Glob Ment Health (Camb). 2024 Mar 21;11:e41. doi: 10.1017/gmh.2024.32 (PMC11018554; doi:10.1017/gmh.2024.32)
Supplement: Peer review history

# Editor report: Recommendation: Psychotherapy training in postgraduate psychiatry training in Nigeria – Are we doing enough? — R0/PR2

**DOI:** 10.1017/gmh.2024.32.pr2

**Affiliations:** University of Zimbabwe Faculty of Medicine, Zimbabwe

Thank you for highlighting the findings on psychotherapy training for psychiatrists in Nigeria. Please state if ethics approval was obtained for this research study.

Please format the tables that are in the manuscript and perhaps use smaller font size for the text in the tables.

Please correct on page 15 paragraph 1. “....sort out...” should be “...sought...”

# Editor report: Decision: Psychotherapy training in postgraduate psychiatry training in Nigeria – Are we doing enough? — R0/PR3

**DOI:** 10.1017/gmh.2024.32.pr3

**Affiliations:** London School of Hygiene & Tropical Medicine, United Kingdom of Great Britain and Northern Ireland

No accompanying comment.

# Editor report: Recommendation: Psychotherapy training in postgraduate psychiatry training in Nigeria – Are we doing enough? — R1/PR5

**DOI:** 10.1017/gmh.2024.32.pr5

**Affiliations:** University of Zimbabwe Faculty of Medicine, Zimbabwe

No accompanying comment.

# Editor report: Decision: Psychotherapy training in postgraduate psychiatry training in Nigeria – Are we doing enough? — R1/PR6

**DOI:** 10.1017/gmh.2024.32.pr6

**Affiliations:** London School of Hygiene & Tropical Medicine, United Kingdom of Great Britain and Northern Ireland

No accompanying comment.

# Editor report: Recommendation: Psychotherapy training in postgraduate psychiatry training in Nigeria – Are we doing enough? — R2/PR8

**DOI:** 10.1017/gmh.2024.32.pr8

**Affiliations:** University of Zimbabwe Faculty of Medicine, Zimbabwe

No accompanying comment.

# Editor report: Decision: Psychotherapy training in postgraduate psychiatry training in Nigeria – Are we doing enough? — R2/PR9

**DOI:** 10.1017/gmh.2024.32.pr9

**Affiliations:** London School of Hygiene & Tropical Medicine, United Kingdom of Great Britain and Northern Ireland

No accompanying comment.

# Editor report: Recommendation: Psychotherapy training in postgraduate psychiatry training in Nigeria – Are we doing enough? — R3/PR11

**DOI:** 10.1017/gmh.2024.32.pr11

**Affiliations:** University of Zimbabwe Faculty of Medicine, Zimbabwe

Thank you for the revisions you. Please may you respond to one of the comments from one of the reviewers.

# Editor report: Decision: Psychotherapy training in postgraduate psychiatry training in Nigeria – Are we doing enough? — R3/PR12

**DOI:** 10.1017/gmh.2024.32.pr12

**Affiliations:** London School of Hygiene & Tropical Medicine, United Kingdom of Great Britain and Northern Ireland

No accompanying comment.

# Editor report: Recommendation: Psychotherapy training in postgraduate psychiatry training in Nigeria – Are we doing enough? — R4/PR14

**DOI:** 10.1017/gmh.2024.32.pr14

**Affiliations:** University of Zimbabwe Faculty of Medicine, Zimbabwe

No accompanying comment.

# Editor report: Decision: Psychotherapy training in postgraduate psychiatry training in Nigeria – Are we doing enough? — R4/PR15

**DOI:** 10.1017/gmh.2024.32.pr15

**Affiliations:** London School of Hygiene & Tropical Medicine, United Kingdom of Great Britain and Northern Ireland

No accompanying comment.

# Editor report: Recommendation: Psychotherapy training in postgraduate psychiatry training in Nigeria – Are we doing enough? — R5/PR17

**DOI:** 10.1017/gmh.2024.32.pr17

**Affiliations:** University of Zimbabwe Faculty of Medicine, Zimbabwe

No accompanying comment.

# Editor report: Decision: Psychotherapy training in postgraduate psychiatry training in Nigeria – Are we doing enough? — R5/PR18

**DOI:** 10.1017/gmh.2024.32.pr18

**Affiliations:** London School of Hygiene & Tropical Medicine, United Kingdom of Great Britain and Northern Ireland

No accompanying comment.
